# Delayed Onset Malignant Hyperthermia after Sevoflurane

**DOI:** 10.1155/2013/712710

**Published:** 2013-05-30

**Authors:** K. Sanem Cakar Turhan, Volkan Baytaş, Yeşim Batislam, Oya Özatamer

**Affiliations:** ^1^Department of Anaesthesiology and Reanimation, Ankara University Medical School, Ankara, Turkey; ^2^Department of Anaesthesiology and Reanimation, Ankara Güven Hospital, Ankara, Turkey

## Abstract

Malignant hyperthermia is a hypermetabolic response to inhalation agents (such as halothane, sevoflurane, and desflurane), succinylcholine, vigorous exercise, and heat. Reactions develop more frequently in males than females (2 : 1). The classical signs of malignant hyperthermia are hyperthermia, tachycardia, tachypnea, increased carbon dioxide production, increased oxygen consumption, acidosis, muscle rigidity and rhabdomyolysis. In this case report, we present a case of delayed onset malignant hyperthermia-like reaction after the second exposure to sevoflurane.

## 1. Introduction

Malignant hyperthermia is characterized by a hypermetabolic response to triggering agents. In this case report, we present delayed onset malignant hyperthermia-like reaction after the second exposure to sevoflurane [1]. 

## 2. Case

An 8-day-old boy was scheduled for choanal atresia evaluation under general anesthesia. Anesthesia induction maintenance was done with sevoflurane 7-8%, after intubation remifentanil 2 *μ*g was given. No muscle relaxant was used. Anesthesia lasted 35 minutes without any problem. One week after this procedure, the patient was scheduled for bilateral nasopharyngeal tube application under general anesthesia with sevoflurane. The procedure ended without any problem. During his followup, the temperature increased to 42.5°C, heart rate increased to 250/min, and respiratory distress developed. Creatinine phosphokinase levels reached 929 IU/L, and hyperpotassemia developed. Blood gas analysis revealed hypoxemia (SO_2_ < 85%), respiratory acidosis (PaCO_2_ > 60 mm Hg) and metabolic acidosis (base deficit > 10 mEq/L). The clinical condition of the patient was thought to be due to malignant hyperthermia, and dantrolene sodium was given orally. After dantrolen sodium, the body temperature minimally decreased, and as the respiratory distress continued, the patient was intubated and mechanical ventilation was started. Dantrolen sodium 2.5 mg/kg was given intravenously with 6-hours intervals for 2 days and his temperature decreased. Following 10-hours period of intubation, the patient was extubated and CPAP was done. 

There was no family history of malignant hyperthermia or disease increasing the susceptibility to malignant hyperthermia in this patient. He was born with cesarean section after 39 weeks of gestation and his birth weight was 4050 and APGAR score was 6/8. Any systemic problems and fetal anomalies were not seen during pregnancy. There were 3 abortuses with unknown etiologies before this pregnancy. After the delivery as the baby had syndromic facial appearance and inadequate spontaneous respiration, he was followed up in the newborn service and positive pressure ventilation was done. Physical examination revealed dysmorphic face, micrognathia, high-arched palate, low-set ears, popeyed appearance, hypertelorism, low-slanting palpebral fissures, cryptrochidism, clinodactyly, and craniosynostosis. There was bilateral hearing loss. 

## 3. Discussion

Malignant hyperthermia is a hypermetabolic response to potent inhalation agents (such as halothane, sevoflurane, and desflurane), succinylcholine, vigorous exercise, and heat. Reactions develop more frequently in males than females (2 : 1). The classical signs of malignant hyperthermia are hyperthermia, tachycardia, tachypnea, increased carbondioxide production, increased oxygen consumption, acidosis, muscle rigidity, and rhabdomyolysis [1]. 

The gold standard for diagnosis of susceptibility to malignant hyperthermia is caffeine-halothane contracture test. However, as this test is not widely available, the diagnosis of malignant hyperthermia can only be made by clinical presentation. Dantrolene sodium is a specific antagonist of the pathophysiologic changes of malignant hyperthermia and when given in the early period it is lifesaving [1, 2]. 

Malignant hyperthermia was suspected with the presence of respiratory acidosis, sinus tachycardia, metabolic acidosis, increased serum creatinine kinase levels, hyperpotassemia, and hyperthermia in our patient and dantrolen sodium was given for treatment. The improvement of the clinical signs after administration of dantrolen sodium suggested that our diagnosis of malignant hyperthermia is correct. However, to confirm the diagnosis, caffeine-halothane contracture test could not be done as this test is not available in our institution. 

In our patient, serum creatinine kinase levels increased only moderately and we thought that moderate increase of serum creatinine kinase level may be due to immediate treatment with dantrolene sodium and rapid improvement of the clinical signs. Also, the young age of the patient may be another factor. As the muscle mass in older children and the adults is relatively greater, serum creatinine kinase levels are expected to be higher in older patients. 

Although sevoflurane is known as a less potent agent triggering malignant hyperthermia, malignant hyperthermia after sevoflurane exposure was reported in the literature. Malignant hyperthermia after sevoflurane exposure is thought to be related to calcium release from sarcoplasmic reticulum [3].

Hsu et al. have reported malignant hyperthermia in a boy aged 3 years and 9 months who was scheduled for Hotz's operation under general anesthesia with sevoflurane and the symptoms of this patient have improved after discontinuation of sevoflurane. Molecular genetic testing identified a novel ryanodine receptor (RYR1) mutation in this patient and this confirmed the malignant hyperthermia susceptibility in this patient [2].

Bonciu et al. have reported malignant hyperthermia in a 7-years old boy who was scheduled for tympanoplasty under general anesthesia with sevoflurane. The patient had a history of anesthesia induction with sevoflurane without any complication. But the maintenance had been done with propofol. It was reported that the clinical symptoms were improved with discontinuation of sevoflurane and increasing the minute ventilation [4]. 

The late onset of malignant hyperthermia is a rare clinical entity. Chen et al. have reported malignant hyperthermia in a 5-years old boy after second exposure to sevoflurane. This patient had general anesthesia with sevoflurane for 2 times with an interval of 2 days [3].

Greenberg et al. have reported malignant hyperthermia in a 6-months old baby with 5q chromosomal deletion scheduled for cleft palate repair under general anesthesia with sevoflurane. The patient had a history of magnetic resonance imaging under general anesthesia with sevoflurane 2 weeks before the surgery and he had had increased temperature and tachycardia but these symptoms reversed spontaneously. After the second exposure to sevoflurane tachycardia, increased temperature, and hypercarbia were seen and these symptoms reversed with treatment with dantrolene sodium. Greenberg et al. reported that this is the first case of malignant hyperthermia in a patient with 5q chromosomal deletion in the literature [5]. 

During his first anesthesia experience, our patient had general anesthesia with sevoflurane and no complication were seen. But 1 week later, after his second exposure to sevoflurane, clinical symptoms of malignant hyperthermia was seen during postoperative period. The late onset of malignant hyperthermia after the second exposure is a rare condition. We thought that this may be related to latent effect of volatile anesthetics on skeletal muscles. 

Claussen et al. have reported malignant hyperthermia following general anesthesia with sevoflurane in a 5-year-old boy who had been anaesthetized two times with halothane without any complication. The timely administration of dantrolene rapidly reversed the life-threatening signs and prevent progression of the disease in this patient [6]. In a similar manner, in our patient we have administered dantrolen sodium in the early period and the symptoms reversed.

Reed et al. have reported on two boys aged 2 and 6 years, respectively, with dysmorphic face, ptosis, downslanting palpebral fissures, hypertelorism, epicanthic folds, low-set ears, malar hypoplasia, micrognathia, high-arched palate, clinodactyly, palmar simian line, pectus excavatum, winging of the scapulae, lumbar lordosis, and mild thoracic scoliosis who present congenital hypotonia, slightly delayed motor development diagnosed as King-Denborough syndrome. Reed et al. have emphasized the fact that patients with King Denborough syndrome may undergo general anesthesia for cryptrochidism and skeletal deformities and the objective must be increasing the awareness of this disorder as these patients are predisposed to developing malignant hyperthermia [7]. 

The preoperative physical examination in our patient also revealed dysmorphic face, micrognathia, high-arched palate, low-set ears, popeyed appearence, hypertelorism, low-slanting palpebral fissures, cryptochirdism, clinodactyly, craniosynostosis, and bilateral hearing loss but definite diagnosis was not done by the genetic department. However malignant hyperthermia during the postoperative period following general anesthesia together with the syndromic appearance made us think this may be King Denborough syndrome. 

Kinouchi et al. reported two cases of malignant hyperthermia triggered by sevoflurane and these patients had no familial susceptibility to malignant hyperthermia. Postoperatively, one of these patients was noted to have downslanting palpebral fissures, micrognathia, low-set ears, and a single crease of the fifth finger and diagnosed as King syndrome which is reported to have association with malignant hyperthermia [8]. 

Although there was no definite diagnosis in the preoperative period, the development of malignant hyperthermia during the postoperative period has made us think of this syndrome as King-Denborough syndrome. The King-Denborough syndrome (KDS) is a congenital myopathy associated with susceptibility to malignant hyperthermia, skeletal abnormalities, and dysmorphic features with characteristic facial appearance. As there is susceptibility to malignant hyperthermia in these patients, it is important to evaluate the clinical signs of King-Denborough syndrome during the preoperative period especially in the newborn. 
